# Novel ideas for the high luminosity phase of the LHC

**DOI:** 10.1140/epjc/s10052-022-10906-9

**Published:** 2022-11-03

**Authors:** Kristin Lohwasser, Matthias Schott

**Affiliations:** 1 Department of Physics, University of Sheffield, Sheffield, UK; 2 Institut für Physik, Universität Mainz, Mainz, Germany

## Abstract

Without doubt, the High Luminosity Large Hadron Collider (HL-LHC) is one of the major projects in particle physics at the high energy frontier in the upcoming decades. Planned to come into operation mid-2027 it will substantially increase the amount of proton-proton collisions at 14 TeV delivered to the LHC experiments with a planned integrated luminosity of 3000 $\mathrm {fb^{-1}}$ for both ATLAS and CMS experiments, 50 $\mathrm {fb^{-1}}$ for LHCb, and 5 $\mathrm {fb^{-1}}$ for ALICE. The delivery of Pb–Pb and p–Pb collisions with integrated luminosities of 13 $\mathrm {nb^{-1}}$ and 50 $\mathrm {nb^{-1}}$ is foreseen, yielding an increase by a factor of up to twenty compared to the currently available data of Run-1 and Run-2.

## Beyond the HL-LHC physics case

It became soon obvious to the particle physics community that an HL-LHC would offer a unique chance to significantly advance our current understanding of particle physics and a large variety of measurements will get into reach for the first time [1, 2]. Examples for the rich physics programme are Higgs decays into $\mu ^+\mu ^-$ and $Z\gamma$ final states and the trilinear Higgs self-coupling, which can be studied in Higgs pair production. Furthermore the measurement of polarised vector-boson scattering will be targeted. The energy reach of inclusive jet and di-jet production in the central rapidity range will be extended by factors of 1.2–1.5. However, ultimately due to the relatively small increase in centre-of-mass energy, most improvements will derive from the huge increase in the size of the data sets and therefore will be – unlike during Run-1 and Run-2 – accumulated over a time scale of several years.

The European Strategy for Particle Physics which was approved by the CERN Council in June 2020 [3] prioritizes the exploitation of the HL-LHC, but it also emphasizes the need to build a vision for the future. The preferred projects are a dedicated lepton collider as Higgs factory for precision studies in parallel to studying the technical and financial feasibility for a next-generation hadron collider. At the same time, the support for neutrino physics in the USA and Japan should be maintained.

Whilst the intention here is not to decrease the engagement with the HL-LHC (quite the opposite), it is still inevitable: Now is the time to start engaging with the new proposed projects and become a leader in one of these new activity areas. Similar tendencies were observed during the last years of running of the LEP, HERA and the Tevatron colliders where collaboration sizes dwindled whilst large data sets were still being recorded which subsequently were analyzed in smaller groups; some analyses were not carried out at all due to the missing person power. The long time scales of the HL-LHC will presumably pose a problem for PhD students who generally need to publish on a timeline of a few years in order to have a chance for a career in particle physics. The less opportunities for publications, the less students will join the experiments and the less person power will be available to sustain the experiment and conduct world-leading measurements. Often this is compensated by recruiting new collaborators into the collaborations which are geographically more dispersed with less means to travel regularly to the experiments. At the same time, travel might become more sparsely available, given immense effort to reduce carbon emissions to limit climate change [4, 5].

To address these challenges we – as a community – need to find and document new ideas for LHC physics during Run-4 and beyond. In particular we need to ask ourselves, which physics measurements can be done beside a handful of flagship analyses? What can be done to maximize the physics output? How can we improve detector performance and optimize the use of resources? What are our long term goals for the collaborations and their culture?

With the Covid-19 pandemic and the related travel restrictions an explosion of experiments with virtual conference formats happened – either forced by the pandemic (as e.g. the International conference of High energy physics - ICHEP2020 in “Prague-on-the-net” or the Large Hadron Collider Physics Conference for which both the $8^{\textrm{th}}$ and $9^{\textrm{th}}$ Edition had to be moved online) or by choice as was the case for “Cosmology from home”^1^ or the Offshell-2021^2^ conference. The latter targeted the particle physics community and was organized to collect novel ideas for the HL-LHC. Participants were required to submit abstracts for conference contributions that would be publishable as original papers (instead of pure proceedings) to increase the motivation for participants to engage in the conference and discuss new ideas. While this concept of accepting only novel and highly innovative contributions is already standard practice in many other fields of sciences, it is a new approach in the area of particle physics, where mainly previously published results are presented. The editorial board of the European Physical Journal C kindly agreed to act as a host journal for the peer-reviewed and innovative submissions to the conference and hence allowed for the first time to probe an alternative approach for conference submissions in the area of particle physics.

The new ideas presented ranged from new searches and measurements during the HL-LHC phase, over innovative analysis methods and tools to new experimental setups at the LHC. In addition, overview talks on the challenges and opportunities from the experimental and theoretical communities have been given and discussed.

The schedule was arranged in two live time slots containing the same contributions with presentations given by authors residing in that particular time zone.^3^ Admittedly though, there was a markedly higher attendance for the European afternoon slot. In addition, there was a virtual poster session added that allowed for natural small-group discussions, connecting only those participants via video and audio whose avatars were in close proximity on a virtual map. There has been some – anecdotal – evidence of people finding collaborators also during virtual workshops and the participation is easier for researchers with less access to travel grants. However, the drawbacks should not be left unmentioned: Actual participation is usually only half or a third of the number of those registered and a virtual meeting cannot fully replace an in-person one. Also organisation becomes markedly difficult with the committee^4^ spread around the globe. Still, of the Offshell-2021 conference one legacy remains: This collection of novel ideas for the future.

## Data Availability

This manuscript has no associated data or the data will not be deposited. [Authors’ comment: This is an editorial with no associated measurement or data analysis, therefore no data will be deposited.]

## Footnotes

1 https://www.cosmologyfromhome.com/.

2 https://indico.cern.ch/event/968055/.

3 Around a third of the presentations was recorded and played by the session moderators.

4 Conference co-organizers were Alberto Belloni, Kingman Cheung, Jan Fiete Grosse-Oetringhaus, Stefania Gori, Kristin Lohwasser, Chilufya Mwewa, Stathes Paganis, Matthias Schott and Mika Anton Vesterinen (in alphabetical order).
